# Respiratory Symptoms and Lung Function among Ethiopian Women in Relation to Household Fuel Use

**DOI:** 10.3390/ijerph17010041

**Published:** 2019-12-19

**Authors:** Mulugeta Tamire, Adamu Addissie, Abera Kumie, Emma Husmark, Susann Skovbjerg, Rune Andersson, Mona Lärstad

**Affiliations:** 1 Department of Preventive Medicine, School of Public Health, Addis Ababa University, Addis Ababa, P.O. Box 9086, Ethiopia; adamuaddissie@gmail.com (A.A.); aberakumie2@yahoo.com (A.K.); 2 Department of Occupational and Environmental Medicine, Institute of Medicine, Sahlgrenska Academy, University of Gothenburg, Medicinaregatan 16A, 41390 Gothenburg, Sweden; mona.larstad@amm.gu.se; 3 Bergsjön Primary Care Center, Rymdtorget 8D, 41519 Gothenburg, Sweden; emma.husmark@gmail.com; 4 Department of Infectious Diseases, Institute of Biomedicine, Sahlgrenska Academy, University of Gothenburg, 41345 Gothenburg, Sweden; susann.skovbjerg@vgregion.se (S.S.); rune.andersson@gu.se (R.A.); 5 Department of Clinical Microbiology, Sahlgrenska University Hospital, Region Västra Götaland, Guldhedsgatan 10A, 41346 Gothenburg, Sweden; 6 Department of Respiratory Medicine and Allergology, Institute of Medicine, Sahlgrenska University Hospital, 41390 Gothenburg, Sweden

**Keywords:** household air pollution, solid fuel, respiratory symptoms, lung function, Africa

## Abstract

Exposure to household air pollution has been linked to chronic obstructive pulmonary disease, respiratory symptoms and reduced lung function. This study aims to assess respiratory symptoms and lung function among Ethiopian women in relation to exposure to HAP. We conducted a cross-sectional study among non-smoking women responsible for household cooking. Data was collected on socio-demographic characteristics, respiratory symptoms and risk factors using a validated questionnaire. Spirometry with reversibility testing was performed according to American Thoracic Society/European Respiratory Society guidelines. We used independent t-test and multivariable logistic regression to compare the means and measure association respectively. A total of 545 women participated in the study out of which 231 (42.3%) performed spirometry with at least three acceptable manoeuvres. Everyone in the rural group and 43% of the urban group were exposed to HAP from solid fuels during cooking. The odds of developing at least one respiratory symptom when compared with those using cleaner fuels are twice as high for women cooking within the living house. We also found significantly lower forced expiratory volume in the first second (FEV1) (L) among solid fuels users compared with cleaner energy users. Given the larger population settlement in the rural areas and the use of solid fuel as the only energy source, there is a higher risk of developing chronic respiratory health problems for those women in Ethiopia.

## 1. Introduction

Nearly three billion people living in low and middle-income countries still cook and heat their homes using solid fuels (i.e., wood, charcoal, dung or crop wastes, collectively referred to as biomass fuels) on open fires or traditional stoves. These practices are inefficient and produce high levels of household air pollution (HAP) including a range of health damaging pollutants such as small soot particles which penetrate deep into the lungs [1,2] and carbon monoxide (CO) with exposures often far exceeding the national standards and international guidelines [3]. The women, especially those responsible for cooking, and their young children, are most heavily exposed as they spend the most time near the domestic hearth [1,4].

According to WHO, over 4 million people a year throughout the world, die prematurely from the exposure to HAP with the overall disease burden in disability adjusted life years (DALYs), exceeding the burden from outdoor air pollution five-fold. In low and middle-income countries, household smoke is responsible for an estimated 3.7% of the overall disease burden [1]. Exposure to HAP has been linked to a range of adverse health outcomes including chronic obstructive pulmonary disease (COPD), airway infections, impaired lung growth, lung cancer as well as cataracts in adults [2,5,6,7,8,9]. It has been suggested that women with domestic exposure to solid fuel combustion may develop COPD with clinical characteristics, an impaired quality of life and increased mortality similar in extent to those of the tobacco smokers [7,10,11]. Other systematic reviews and meta-analysis studies have also confirmed that exposure to solid fuel smoke is consistently associated with COPD [11,12,13].

It is often assumed that people living in lower economic areas have a higher risk for adverse health effects associated with household environmental exposures [14]. Like other low-income countries, Ethiopian household energy use is mainly dependent on solid fuels with over 90% using wood as their main energy source for cooking, though high urban-rural discrepancy exists [15]. Studies conducted in different parts of the country reported the level of particulate matters and other pollutants at homes using solid fuels and traditional stoves far exceeding the international standards, thus posing a further public health concern in the region [15,16,17]. As many as 97% of the households in southern Ethiopia use wood for cooking and 56% have indoor kitchens not separated from the rest of the house [15]. In the study from Butajira area of the same region, high indoor nitrogen dioxide (NO_2_) levels from biomass fuels were measured [17]. Meanwhile, the use of biomass fuels was found to be associated with high levels of HAP in the slum areas of Addis Ababa, where biomass fuel is predominantly used [16].

The vast majority of the population in Ethiopia rely on the use of solid fuel and are daily heavily exposed to HAP; no prior study has involved women with evaluation of symptoms and lung function. Here, our main aim was to assess respiratory symptoms and lung function in Ethiopian women in relation to exposure to HAP. We compared the status of mothers in urban Addis Ababa to mothers living in rural Ethiopia, respectively. In addition, we compared the status of mothers using solid fuels with those using cleaner fuels, regardless of area. Data will add value in providing relevant information for planning and implementation of intervention activities by the government and non-governmental organisations.

## 2. Materials and Methods

### 2.1. Study Design and Period

A descriptive, comparative cross-sectional study was carried out from March to August 2016.

### 2.2. Study Area

The study was conducted in Addis Ababa, the capital city of Ethiopia and the rural area of Butajira. Addis Ababa is situated in the central part of Ethiopia at an altitude of approximately 2355 metres above sea level. The rural area of Butajira in the Gurage zone of the Southern Nations and Nationalities and Peoples Region (SNNPR), is approximately 136 km south of Addis Ababa at a moderate altitude (2131 m) similar to Addis Ababa. One urban and nine rural kebeles (the lowest administrative level) of the area have been the site of the demographic surveillance system (DSS) of Addis Ababa University Rural Health Programme, since 1987 [18]. The vast majority of households in the Butajira area exclusively burn biomass materials as a main source of energy for cooking and heating on open fires mostly using traditional stoves while some use locally made improved cook stoves [19]. The women spend most of the day in the room with a lit fire, whereas tobacco smoking is uncommon among adult women in the area and across the country [20].

### 2.3. Study Population

Mothers with a child below two years of age at the time of data collection, who were willing to take part in the study, were included because of a simultaneous study of their children. They were recruited using systematic sampling technique at four purposively selected health centres that were located in the slum areas of Addis Ababa (*n* = 266) and four rural kebeles of Butajira (*n* = 279). While mothers who came with their children for the vaccination service at the health centres were included in the urban setting, the rural mothers, with children less than two years of age, were recruited from the villages at Butajira because health extension workers gave most children vaccinations in their villages not at health centres. As the expected sample size for all visiting the health centres during the data collection and those available in the rural areas were nearly equal, all who fulfilled the inclusion criteria were included. Tobacco smoking women as well as mothers who were not permanent residents of the catchment areas were excluded from this study. For examination with spirometry, exclusion criteria were a recent heart attack, recent ophthalmic, thoracic or abdominal surgery, pregnancy and acute disorders affecting test performance.

### 2.4. Data Collection

Three data collection methods were employed: interviewer administered questionnaire, anthropometry and spirometry measurements.

#### 2.4.1. Questionnaire

Face-to-face interviews were conducted using a structured questionnaire prepared in English adopted from the Medical Research Council (MRC) questionnaire on respiratory symptoms, UK [21] with contextual modification. It was translated to Amharic (national language) with back translation to check for consistency. The questionnaire contained socio-demographic characteristics, questions regarding cooking conditions such as type of fuels, housing and ventilation and respiratory symptoms such as cough, wheezing, phlegm, shortness of breath and irritation of the nose in the last 12 months.

#### 2.4.2. Anthropometry

Standing height and weight of the mothers were taken in wearing light clothing and barefoot using standard weight and height measure. Body mass index (BMI) was calculated from the height and weight (kg/m^2^). We used two weight scales, the data collectors took their own weights on both each morning to check for accuracy.

#### 2.4.3. Spirometry and Quality Control

Spirometry with reversibility testing was performed according to American Thoracic Society/European Respiratory Society (ATS/ERS) guidelines [22] using a portable spirometer (EasyOne^TM^ World Spirometer; ndd Medizintechnik AG, Zürich, Switzerland), utilising robust ultrasonic measurement technology. We used Easyware 2013 Version 2.25.0.0 software (ndd Medizintechnik AG, Zürich, Switzerland) to transfer the data to a computer. Lung function measurements included forced vital capacity (FVC), forced expiratory volume in the first second (FEV1) and the ratio FEV1/FVC were calculated as well as reversibility of FEV1. Peak expiratory flow (PEF) was also measured. Predicted values were calculated using global lung initiative (GLI) reference values, using Caucasian as the reference group because of evidence from previous research of Eurasian ancestry [23] and historical ethnicity classification of the Horn of Africa, no reference group is available for this region. Participants were given four actuations (in total 800 µg) of short-acting β_2_-agonist salbutamol administered with an Easyhaler (to avoid possible infections with a spacer) and spirometry was repeated after 15–30 min. We only included participants with at least three, but not more than eight, technically acceptable breathing manoeuvres as per the acceptability and repeatability criteria recommended by the ATS/ERS task force to obtain good quality test sessions [22,24]. Flow volume calibration was performed with a 3 L calibration syringe with ±3% accuracy from the manufacturer; and results were always well within the approved calibration limits. The EasyOne spirometer remains consistently accurate over the years; it has a demonstrated volume accuracy of ±3% over at least four years with no significant nonlinearity [25]. All spirometric measures were carried out with the subject wearing a nose clip and using a disposable mouthpiece. Single-packed, disposable mouthpieces (ndd EasyOne Spirettes; Bluebird Medical AB, Kungsbacka, Sweden) assured perfectly hygienic conditions for each participant.

Trained and experienced graduate nurses and health officers from Butajira Hospital and health centres of Addis Ababa were involved in conducting structured interviews, performing the lung function tests and clinical assessments after additional two days training with a pre-test before actual data collection. Quality controls were performed continuously during the data collection.

### 2.5. Operational Definition

Cough: We considered someone to have cough if their answer was “yes” to at least one of the following four questions. (i) Cough first thing in the morning; (ii) cough during the day or night; (iii) cough as much as four to six times a day in a week; or (iv) cough for most of the day for as much as three consecutive months during a year.

Phlegm: We considered someone to have phlegm if they usually bring up phlegm from their chest (deep down in her lungs) first thing in the morning or during the day in the last 12 months.

Breathlessness: We considered some to have breathlessness if troubled by a shortness of breath when hurrying on level ground, walking up a slight hill, when walking at their own pace on the level ground and stop for breath after a few min/100 m or breathless when dressing/undressing or cooking.

Wheezing: We considered someone to have wheezing if her chest (lungs) ever sound wheezy (whistling sound).

At least one respiratory symptom: At least one of either cough, phlegm, wheezing, breathlessness or nose irritation.

Permanent opening: Small permanent opening near the end wall in the cooking room.

### 2.6. Data Management and Statistical Analyses

Data entry and cleaning using statistical software for epidemiology EPI-Info Version 3.5.4 (Centres for Disease Control and Prevention (CDC); Atlanta, GA, USA) were made and exported to IBM statistics Version 24 SPSS (SPSS Inc., Chicago, IL, USA) for analysis. After visualizing the general features of the data, descriptive statistics such as mean and standard deviation for continuous variables and frequency and percentage for categorical variables were determined separately for urban and rural participants. Chi-square test of independence was used to test whether or not a statistically significant relationship existed between the place of residence and the following symptoms: cough, phlegm, wheeze, breathlessness, irritated nose and eye irritation. Not to violate assumptions of Pearson Chi-square test, we also checked for Fisher exact test for cells with an expected count less than five. Independent student’s *t*-test was used to compare the values of continuous variables. Bivariate logistic regression was carried out to determine the distribution of the study subjects by independent variable of interest and to see crude association; whereas multivariable logistic regression analysis was used using adjusted odds ratio. Those variables found to be associated with the dependent variable in the bivariate analysis or had *p*-value below 0.25 were exported to multivariable logistic regression analysis to evaluate relative effect of solid fuel use by adjusting for other exposure factors. For all tests, the *p*-value was set to <0.05 to determine significance.

### 2.7. Ethical Approval

Ethical approval was obtained from the Institutional Review Committee of the College of Health Sciences of Addis Ababa University and the National Research Ethics Review Committee (NRERC, 3.10/168/2016), Ministry of Science and Technology, Ethiopia. Permission to conduct the research was secured from respective organizations at both settings. All mothers were asked to give informed consent whereas confidentiality and anonymity were kept throughout the study. Mothers who had acute and chronic respiratory symptoms were advised to visit a clinician for appropriate diagnosis and treatment.

## 3. Results

### 3.1. Background Information

Table 1 shows the basic characteristics of the participants. A total of 545 mothers were enrolled in this study with almost equal proportion from the urban 49% and rural 51% settings. The overall mean age of the mothers was 30.3 years; the mean age of the mothers in the rural area (32.2 years) being significantly higher than that of the urban group (28.4 years) while the body mass index (BMI) of the urban mothers was higher compared with their counters. By education, 52% of the rural mothers had not attended school at all, compared with 10% of the urban mothers. Family size in the rural area was larger—67% consisted of five or more family members, compared with 27% of the urban families. The majority of all mothers were homemakers. As many as 60% of the urban participants were brought up in rural parts of Ethiopia.

### 3.2. Housing and Cooking Practices

All mothers from the rural area used solid fuel (primarily wood) for food cooking compared with 43% in the urban group. Both the mean frequency of cooking and the estimated average duration of a single cooking episode were higher in the rural settings than in the urban area. More than half the mothers from both settings reported a window in the cooking area as the means of ventilation. Second-hand tobacco smoke was significantly lower in the rural setting (Table 2).

### 3.3. Respiratory Symptoms

As shown in Table 3, a cough, wheeze, irritation of nose or presence of at least one respiratory symptom or irritation of the eyes during the last 12 months were significantly more often reported by the rural mothers compared with the urban group. Among the 116 mothers who had a cough, a significantly higher proportion of the rural mothers reported chronic symptoms, i.e., coughing for three or more months, compared with the urban mothers.

### 3.4. Prevalence of Symptoms Related to Fuel Type and Ventilation in the Cooking Area

**Figure 1:**
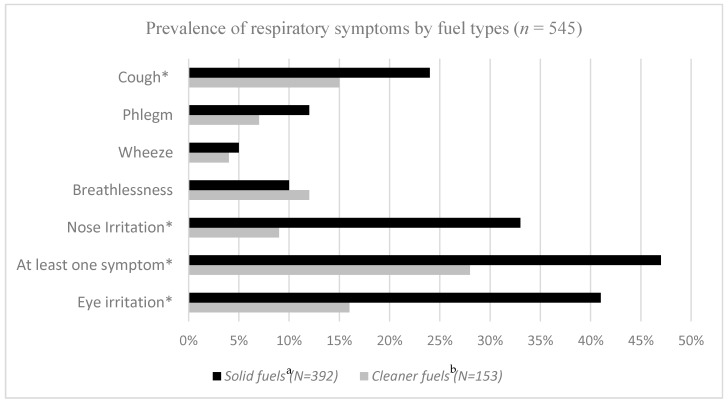
Prevalence (in %) of respiratory symptoms by fuel types in all participants (*n* = 545). * significant at significance level of 0.05. ^a^ wood, charcoal, dung or crop wastes, ^b^ Liquefied petroleum gas or electricity.

We compared the prevalence of symptoms related to fuel type in the whole group, i.e., in both rural and urban women, regardless of the area. The distribution of each symptom related to type of fuel is presented in Figure 1, showing that all symptoms, except wheeze and breathlessness, were significantly more prevalent among women using solid fuels (i.e., wood, charcoal, dung or crop wastes) compared with those using cleaner fuels (liquefied petroleum gas (LPG) or electricity) in the Chi-square test. Irritation of the eyes and nose affected as twice as many of the solid fuel users compared with those using cleaner energy while cough and phlegm also affected a higher proportion of the mothers using solid fuels.

**Figure 2:**
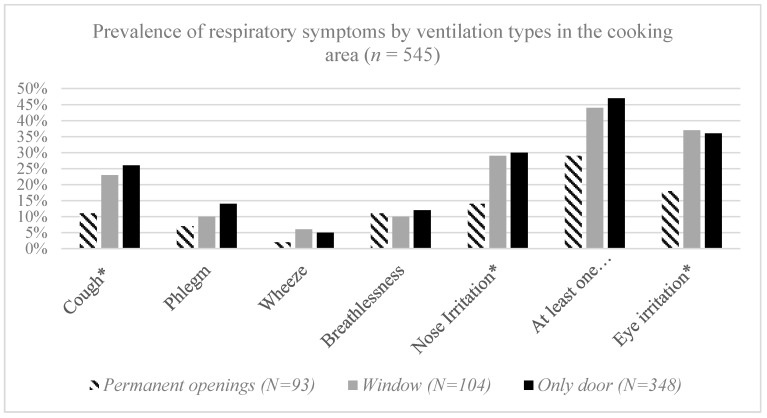
Prevalence of respiratory symptoms by ventilation types in the cooking area among the participants (*n* = 545). * significant at significance level of 0.05.

We also calculated the prevalence of respiratory symptoms related to the type of ventilation in the cooking area for all participants. Accordingly, the proportion of mothers with symptoms was similar to those having only a door compared with those having an additional window as the means of ventilation. However, the existence of a permanent opening in the cooking room was related to a lower prevalence of reported respiratory symptoms (*p*-value < 0.001) (Figure 2).

### 3.5. Factors Associated with Occurrence of at Least One Respiratory Symptom

In the multivariable logistic regression model, we included those variables with a significance level below 0.25 in the bivariate model. Accordingly, fuel type, cooking place, ventilation in the cooking area, exposure to passive smoking at home, place of residence and the age of the mother were included. Assuming the use of cleaner fuels is not risky whether the family cook inside the living house or outside, we combined the cooking place and fuel type used to control for it. Neither cooking frequency nor duration were associated with the occurrence of any respiratory symptom with the required significance level in the bivariate regression.

The prevalence of at least one respiratory symptom (either cough, phlegm, wheeze, breathlessness or nose irritation) was significantly higher among mothers cooking in the living house using solid fuel (Table 4). Those mothers who cook inside the living house using solid fuels, had 1.89 times higher odds of developing at least one respiratory symptom compared with those using the cleaner fuels (OR 1.89; 95% CI 1.11–3.24). Having a permanent opening as ventilation in the cooking area was found to be protective in the bivariate analysis though its effect was only slightly attenuated in the multivariable regression model.

### 3.6. Spirometry Measurements

Of the total, 394 (72.3%) mothers performed at least three good manoeuvres on the spirometry test but only 231 (42.3%) performed with acceptable manoeuvres as per ERS/ATS criteria. The results of the spirometry tests are presented by the place of residence and fuel type used respectively (Table 5 and Table 6). Among all mothers, 28 (12.1%) and 57 (24.2%) had FVC and FEV1 percent predicted below 80%, respectively. We found statistically significant differences in terms of FVC (L), FEV1 (L) and FEV1 percent predicted between the two settlements; all measures were lower among the rural groups. In addition, there was increased improvement in FEV1 in the post bronchodilator test, indicating reversibility among the rural mothers, though it was not statistically significant.

We also found significantly lower FEV1 (L) among solid fuels users compared with cleaner energy users (2.53 versus 2.64, *p* = 0.039). The FVC (L) and FEV1/FVC ratio were also slightly lower among the same group, although there was no statistical significance. Similar to the rural-urban distribution, there were eight (3.5%) mothers, equally from both groups, with pre-bronchodilator FEV1/FVC ratio below 0.7 and FEV1 was less than 80% of that predicted, therefore, suspected to have obstructive airflow impairments (COPD or asthma). Among them, four (1.7% of the total) showed improvement in FEV1 > 200 mL and FEV1 > 12% following bronchodilator administration indicating asthma, while the same number did not and could thus be COPD cases. In addition, we calculated the proportion of women with FEV1/FVC below LLN (lower limit normal). Accordingly, there were eight women with the ratio below LLN or with FEV1/FVC below fifth percentile; however, there was no statistically significant difference by fuel type used.

## 4. Discussion

We found an increased prevalence of respiratory symptoms and lower FVC, FEV1 and FEV1 percent predicted lung function indices among the rural mothers and solid fuel users. A higher prevalence of respiratory symptoms in our study among mothers using solid fuel for cooking was consistent with studies from Cameroon [26], Bangladesh [27] and Nepal [28]. However, the result was not consistent with a study from Peru, which suggested that chronic bronchitis appeared to be as much of a problem in urban centres as it was in rural [29]. Another study from China reported a higher prevalence of respiratory symptoms in Beijing, the capital city, compared with a rural area and smaller city [30]. This might be linked with tobacco smoking, outdoor air pollution and occupational exposures.

Both the prevalence of the respiratory symptoms and the exposure to the solid fuels were high compared with another study from Rwanda [31]. Except for breathlessness, which was found to be higher in that study, the prevalence of other respiratory symptoms among never-smokers were nearly doubled in this study when compared with other Sub-Saharans studies [31,32,33]. Not only did all rural women in the present study use solid fuel as the only energy source, most of them also had a larger family size, poor ventilation, animals in the same house and performed cooking in the main living room. This might receive attention as more than 80% of the total population of the country reside in the rural areas [34] and rely on solid fuel as the source of energy. The overcrowded living situations in small tukul houses in rural communities [35] might also aggravate the respiratory health problems in the group. Comparing our findings, the level of exposure to biomass fuel was reported to be higher in an urban setting of Malawi [32]. The difference could be explained by the different urban settings assessed; here we included women in the capital city of Addis Ababa, while a comparatively smaller urban setting was assessed in Malawi. However, the prevalence of cough, phlegm and wheeze were found to be lower in the present study than recently found in Tanzania [36]. This might be linked with the higher prevalence of smokers and older age range of the participants in that study.

As already mentioned in our study, women using solid fuel for cooking in the living house showed 1.89 times higher odds of having at least one respiratory symptom compared with cleaner energy users, which is consistent with previous studies [26,27,31,33,37]. Meanwhile, the distribution of at least one of the respiratory symptoms was nearly equal among those having a window in the cooking area and those having only the door as a means of ventilation. This might be related to the behaviour of the mothers and cultural aspects of not opening the windows during cooking as explored in our previous qualitative study in the same area [19]. A study from China also reported the existence of never-opened windows, which were significantly improved by behavioural intervention [38]. In fact, the existence of permanent openings in the cooking area contributed to a reduction of the symptoms in our study.

In this study, all spirometry test results were lower among rural mothers and solid fuel users compared with their counters, though FEV1, FVC and FEV1% predicted were significantly lower among the rural groups compared with the urban and only FEV1 was significantly lower among those using solid fuel. Similarly, lower FVC and FEV1 were reported among women using biomass fuel in rural India [39] where there were no mothers with a post-bronchodilator FEV1/FVC less than 0.70 used to confirm the presence of persistent airflow limitation according to the global initiative for chronic obstructive lung disease (GOLD) [40]. However, there are some studies showing the use of pre-bronchodilator FEV1/FVC less than 0.70 could also be used though the accuracy was found to be lower than that of the post-test [41,42]. Accordingly, there were eight mothers (3.5%) with FEV1/FVC below 0.7 and FEV1 was less than 80% of that predicted in the pre- bronchodilation test; therefore, suspected to have obstructive airflow impairments, possibly COPD or asthma, though the distribution was neither different by residence nor fuel types used. In this study, the number of women with FEV1/FVC below LLN were also the same as that of the pre-test ratio. Nevertheless, previous studies have reported a higher prevalence of chronic respiratory diseases including COPD among solid fuel users [7,32,43]. Meanwhile, the occurrence of more episodes of respiratory symptoms and significantly reduced FEV1 among the solid fuel users in our study could indicate the risk of developing those problems in the future. This is supported by a previous finding in Mexico, which found the decline in FEV1 was slower among patients with biomass exposure compared with those who had tobacco exposure [44]. There were four (1.7% of the total), who did not show improvement in FEV1 following bronchodilator administration and could thus be COPD cases. This figure is lower than a previous study among non-smokers in Tunisia although there were male participants in that study and the age range exceeded 40 years where the frequency of chronic obstructive disease could possibly be higher [7].

The absolute values of FVC and FEV1 in litres are nearly equal to a previous studies from Ethiopia [45,46] and another study from Guatemala with a similar age range [47] though per cent predicted FEV1 was lower (82%) in the study from Ethiopia. The most reasonable cause for the higher predicted value in our study could be the reference population used. In addition, in the Guatemalian study the measurements of the variables were derived from a single forced expiratory spirogram recorded by a Vitalograph spirometer, while our study used the best measure after a minimum of three acceptable manoeuvres.

This is, to our knowledge, the first study that measured both housing and cooking practices, fuel use, respiratory symptoms as well as lung function at the community level in Ethiopia. This information adds to the growing body of data regarding HAP effects on respiratory symptoms and lung function. This study used self-reported cooking practices and respiratory symptoms over the last 12 months and could be subjected to recall bias. The use of cross-sectional study poses an inability to see temporal relationship, but in this study, only one direction of causality is biologically plausible. A smaller number of the participants did the spirometry in a standing position. However, the result did not differ from the results in a sitting position because the amount of air a person can exhale will be equal as long as the individual is sitting up straight and there are no restrictions [48]. In addition, it would have been valuable to include older women with higher total HAP exposure time in order to assess HAP effects on lung function. The lack of spirometry prediction equation for Ethiopia could affect the predictions.

## 5. Conclusions

We found a higher prevalence of respiratory symptoms and a slightly reduced lung function test among the rural women when compared with the urban group. Given the larger population settlement in the rural parts and the use of solid fuel as the only energy source, there is a higher risk of developing chronic respiratory health problems for those women in Ethiopia in addition to their burden of fuel collection. The results of spirometry measurements also support the potential likelihood for the risks in older age unless interventions are made to protect the mothers. Higher odds of respiratory symptoms among mothers, who cook inside the living house using solid fuel, indicate the need for a nationwide awareness-creation on the use of cleaner energy or having a separate kitchen. Behavioural intervention by health education is demanded to influence opening existing windows during cooking. The Government and other concerned organisations should also intervene in making cleaner fuel alternatives available to the public. We would like to recommend further spirometry studies at population level to derive prediction equations for the country.

## Figures and Tables

**Table 1:** Socio-demographic characteristics of the study population, Ethiopia, 2016.

Characteristics (Descriptions)		Urban (*n* = 266)	Rural (*n* = 279)	All (*n* = 545)	*p*-Value
Age (years)	Mean (SD)	28.4 (5.3)	32.2 (6.2)	30.3 (6.1)	<0.001
Height (cm)	Mean (SD)	157.9 (6.7)	159.0 (6.7)	158.5 (6.7)	<0.001
Weight (kg)	Mean (SD)	58.1 (11.1)	50.7 (7.4)	54.3 (10.1)	<0.001
BMI (kg/m^2^) Mean (SD)		23.3 (3.8)	20.1(2.7)	21.6 (3.6)	<0.001
Education *n* (%)	No education	27 (10.2)	144 (51.6)	171 (31.4)	<0.001
	Elementary school	102 (38.3)	113 (40.5)	215 (39.4)	
	High school	83 (31.2)	19 (6.8)	102 (18.7)	
	College and above	54 (20.3)	3 (1.1)	57 (10.5)	
Employment *n* (%)	Employed	49 (18.4)	0	49 (9.0)	<0.001
	Homemaker	189 (71.1)	161 (60.5)	350 (64.2)	
	Merchant	28 (10.5)	8 (2.9)	36 (6.6)	
	Farmer	0	110 (39.4)	110 (20.2)	
Family size *n* (%)	Two to three	113 (42.5)	47 (16.8)	160 (29.4)	<0.001
	Four	82 (30.8)	46 (16.5)	128 (23.5)	
	Five	43 (16.2)	51 (18.3)	94 (17.2)	
	Six and above	28 (10.5)	135 (48.4)	163 (29.9)	
Brought up *n* (%)	Urban	129 (48.5)	13 (4.7)	142 (26.1)	0.001
	Rural	137 (59.5)	266 (95.3)	403 (73.9)	

**Table 2:** Housing, cooking practices and fuel use among urban and rural women in Ethiopia, 2016.

Variables		Urban (*n* = 266)	Rural (*n* = 279)	Total (*n* = 545)	*p*-Value
Cooking in the living room, *n* (%)		209 (78.6)	225 (80.6)	434 (79.6)	<0.001
Cooking in separate kitchen, *n* (%)		57 (21.4)	54 (19.4)	111 (20.4)	0.548
Mean frequency of cooking/day (SD)		1.9 (0.8)	2.5 (0.8)	2.2 (0.8)	<0.001
Average minutes/cooking (SD)		67.4 (25.3)	76.4 (25.4)	72.2 (25.7)	<0.001
Second hand tobacco smoke at home (spouse), *n* (%)		23 (8.6)	11 (3.9)	34 (6.2)	0.023
Fuel type, *n* (%)	Solid fuel ^a^	113 (42.5)	279 (100)	392 (71.9)	<0.001
	Cleaner fuel ^b^	153 (57.5)	0	153 (28.1)	
Ventilation type in the cooking area, *n* (%)	Permanent opening	71 (26.7)	22 (7.9)	93 (17)	<0.001
	Window	151 (56.8)	197 (70.6)	348 (63.9)	
	Only door	44 (16.5)	60 (21.5)	104 (19.1)	

^a^ Wood, charcoal, dung or crop wastes, ^b^ Liquefied petroleum gas or electricity.

**Table 3:** Prevalence of respiratory symptoms of the participants during the last 12 months.

Symptoms	Urban (*n* = 266) *n* (%)	Rural (*n* = 279) *n* (%)	All (*n* = 545) *n* (%)	*p*-Value
Cough	45 (16.9)	71 (25.4)	116 (21.3)	0.016
Cough > 3 months	4 (8.9)	19 (26.7)	23 (19.8)	0.004
Phlegm	22 (8.3)	34 (12.2)	56 (10.3)	0.132
Wheeze	6 (2.3)	21 (7.5)	27 (5.0)	0.005
Breathlessness	30 (11.3)	25 (9.0)	55 (10.1)	0.369
Irritation of nose	35 (13.2)	110 (39.4)	145 (26.6)	<0.001
At least one respiratory symptom *	86 (32.3)	142 (50.9)	228 (41.8)	<0.001
Irritation of eye	56 (21.1)	130 (46.6)	186 (34.1)	<0.001

Data are presented as *n* (%). * At least one of either cough, phlegm, wheeze, breathlessness or nose irritation.

**Table 4:** Presence of at least one respiratory symptom in relation to housing and cooking practices among all participants (*n* = 545).

Variables	At Least One Respiratory Symptom	Crude Odds Ratio (OR)	Adjusted Odds Ratio (AOR)	*p*-Value
	Yes/No (%)			
Fuel type and cooking place				
Solid fuel inside living house	161/313 (51)	2.71 (1.78–4.10)	1.89 (1.11–3.24) *	0.019
Solid fuel in separated kitchen	24/79 (30)	1.12 (0.62–2.02)	0.83 (0.42–1.67)	0.612
Cleaner energy (both places)	43/153 (28)	1	1	
Ventilation (in cooking area)				
Permanent opening (any)	27/93 (29)	0.5 (0.3–0.8)	0.66 (0.35–1.23)	0.198
Window	152/348 (44)	0.9 (0.6–1.4)	0.88 (0.56–1.39)	0.604
Only door	49/104 (47)	1	1	
Second-hand tobacco smoke				
Yes	17/34 (50)	1.42 (0.71–2.85)	1.53 (0.74–3.15)	0.247
No	211/511 (41)	1	1	
Area (Place of residence)				
Urban	86/266 (32.3)	0.46 (0.33–0.65)	0.65 (0.40–1.07)	0.091
Rural	142/279 (50.9)	1	1	
Age	(Continuous)	1.03 (1.00–1.06)	1.01 (0.98–1.04)	0.404

* significant at significance level of 0.05.

**Table 5:** Spirometry (measurements meeting ERS/ATS criteria) using mean (SD) of urban (Addis Ababa) and rural (Butajira) women (*n* = 231).

Pre-Bronchodilator	Urban (*n* = 126)	Rural (*n* = 105)	*p*-Value
FVC, L	3.17 (0.41)	3.05 (0.43)	0.039
FVC% predicted	94.03 (10.7)	91.84 (10.5)	0.121
FEV1, L	2.62 (0.39)	2.49 (0.39)	0.017
FEV1% predicted	89.47 (11.1)	86.42 (11.1)	0.039
FEV1/FVC	0.83 (0.05)	0.81 (0.06)	0.091
PEF (L/min)	6.45 (1.40)	6.34 (1.49)	0.598
**Post-Bronchodilator**	***n* = 110**	***n* = 87**	
FVC, L	3.14 (0.48)	3.09 (0.44)	0.406
FEV1, L	2.68 (0.41)	2.63 (0.37)	0.311
FEV1 reversibility (%)	2.85 (5.24)	4.46 (7.31)	0.070
FEV1/FVC	0.86 (0.05)	0.85 (0.05)	0.486
PEF (L/min)	6.56 (1.72)	6.93 (1.25)	0.095

FVC: forced vital capacity, FEV1: forced expiratory flow in 1 s, PEF: peak expiratory flow.

**Table 6:** Spirometry (measurements meeting ERS/ATS criteria) using mean (SD) of solid fuels and cleaner energy using women.

Pre-Bronchodilator	Clean Energy (*n* = 73)	Solid Fuel (*n* = 158)	*p*-Value
FVC, L	3.20 (0.44)	3.08 (0.41)	0.060
FVC% predicted	94.21 (11.1)	92.49 (10.5)	0.259
FEV1, L	2.64 (0.42)	2.53 (0.37)	0.039
FEV1% predicted	89.63 (11.2)	87.37 (11.2)	0.154
FEV1/FVC	0.83 (0.06)	0.82 (0.05)	0.233
PEF (L/min)	6.39 (1.43)	6.41 (1.45)	0.927
**Post-Bronchodilator**	***n* = 62**	***n* = 135**	
FVC, L	3.20 (0.57)	3.08 (0.41)	0.101
FEV1, L	2.73 (0.45)	2.63 (0.35)	0.127
FEV1 reversibility (%)	2.52 (5.86)	4.04 (6.42)	0.113
FEV1/FVC	0.85 (0.04)	0.85 (0.04)	0.561
PEF (L/min)	6.62 (1.63)	6.76 (1.50)	0.533

FVC: forced vital capacity, FEV1: forced expiratory flow in 1 s, PEF: peak expiratory flow.
